# Supplementary material for: Safety Profile of a Virus-Like Particle-Based Vaccine Targeting Self-Protein Interleukin-5 in Horses
Source: Vaccines (Basel). 2020 May 9;8(2):213. doi: 10.3390/vaccines8020213 (PMC7349629; doi:10.3390/vaccines8020213)
Supplement: Supplementary file 1 [file vaccines-08-00213-s001.pdf]

vaccines

MDPI

Supplementary Materials:

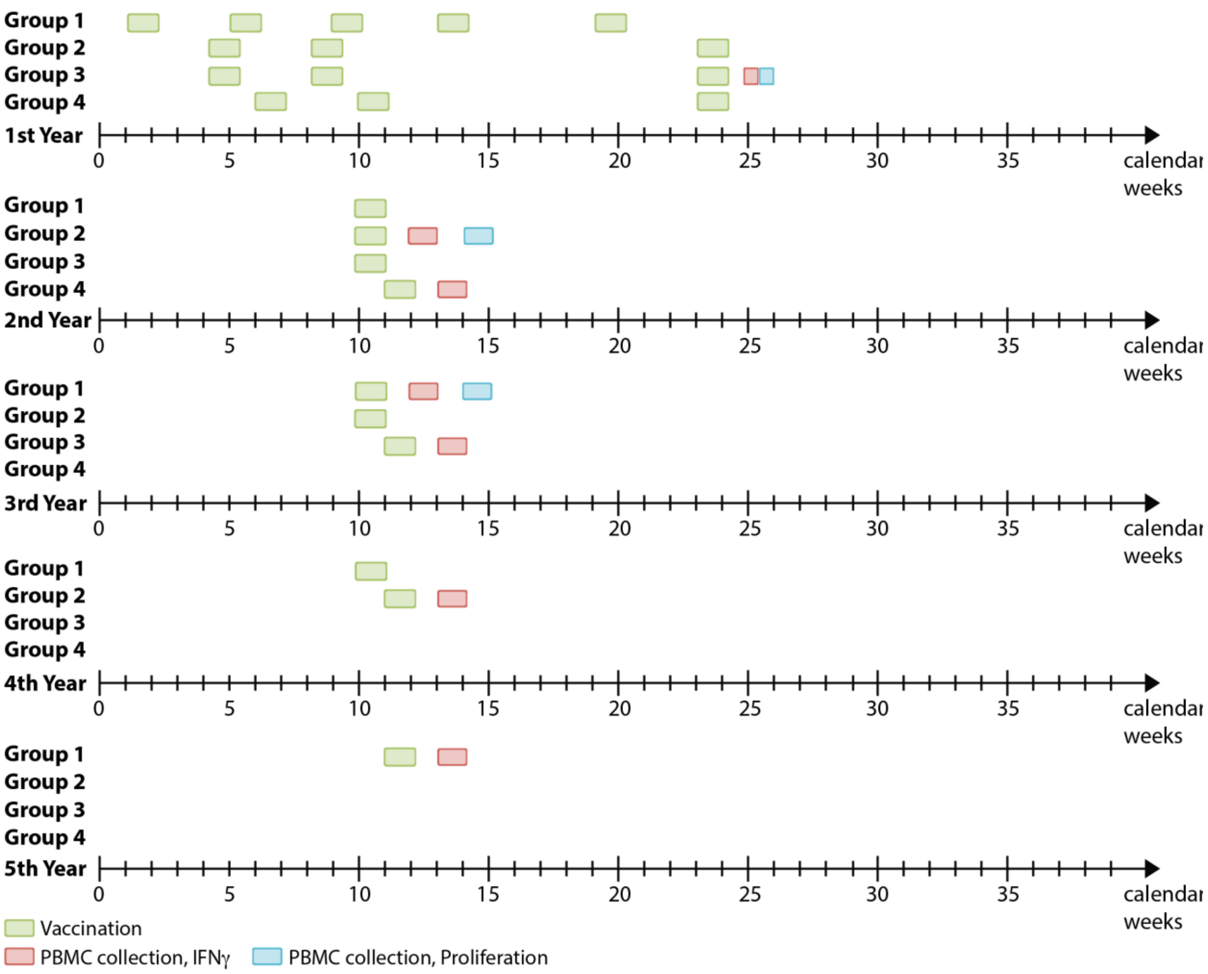

Figure 1. Vaccination regimen. The horses (group 1, 2, 3, 4) were vaccinated (green box) in the first treatment year (1st Year) by a basic vaccination regimen and received a single booster in all follow-up years (2nd, 3rd, 4th, 5th year) in spring before the *Culicoides* season started. Bleedings for IFNγ (red box) and for proliferation (blue box) occurred two or respectively four weeks post boost.

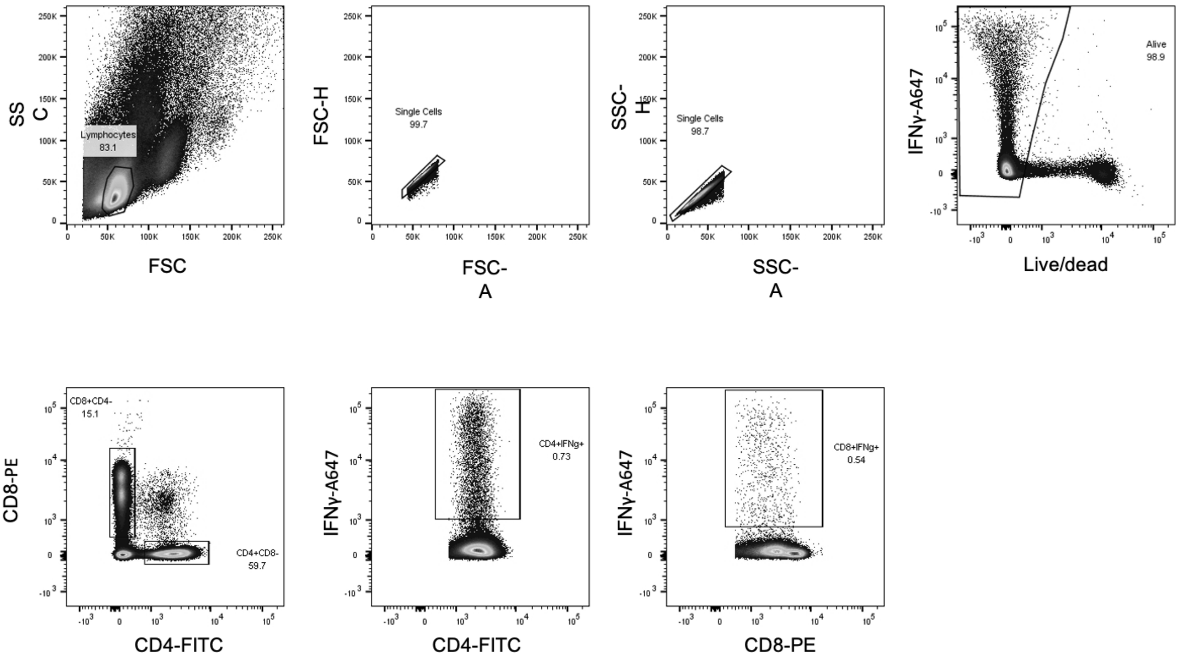

Figure 2. Gating strategy for IFNγ production of $CD4^+$ and $CD8^+$ T cells.

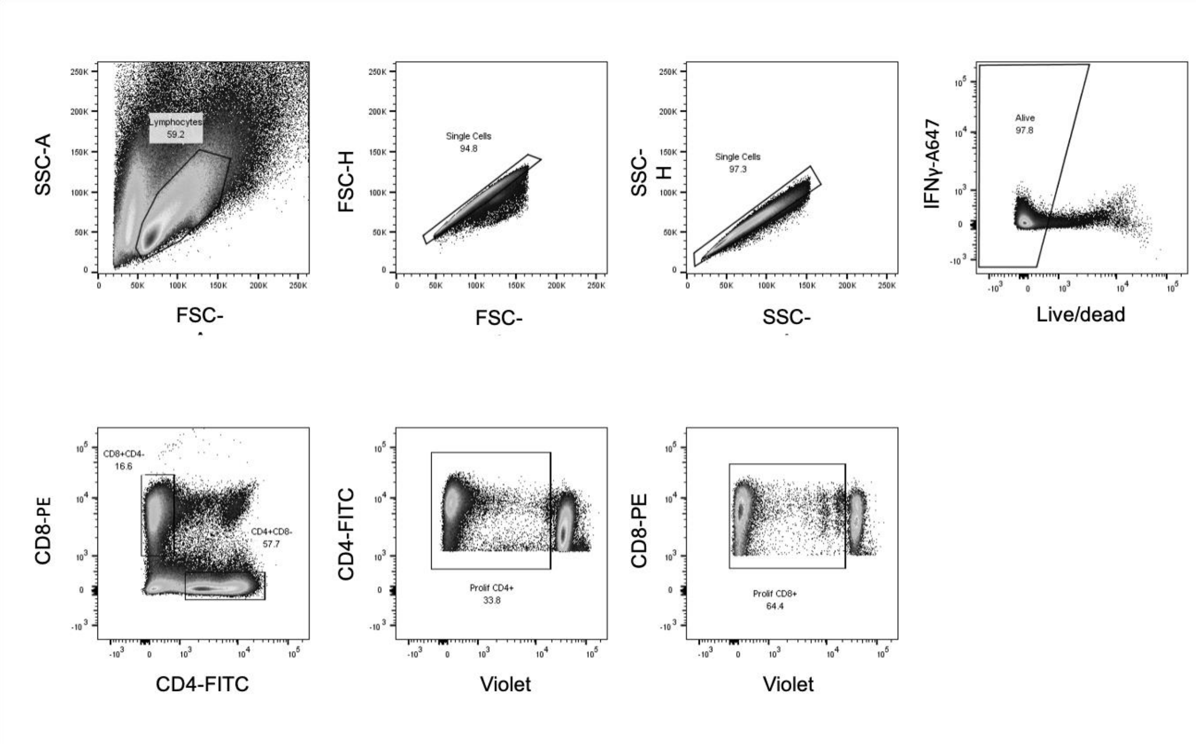

Figure 3. Gating strategy for proliferation of $CD4^+$ and $CD8^+$ T cells.

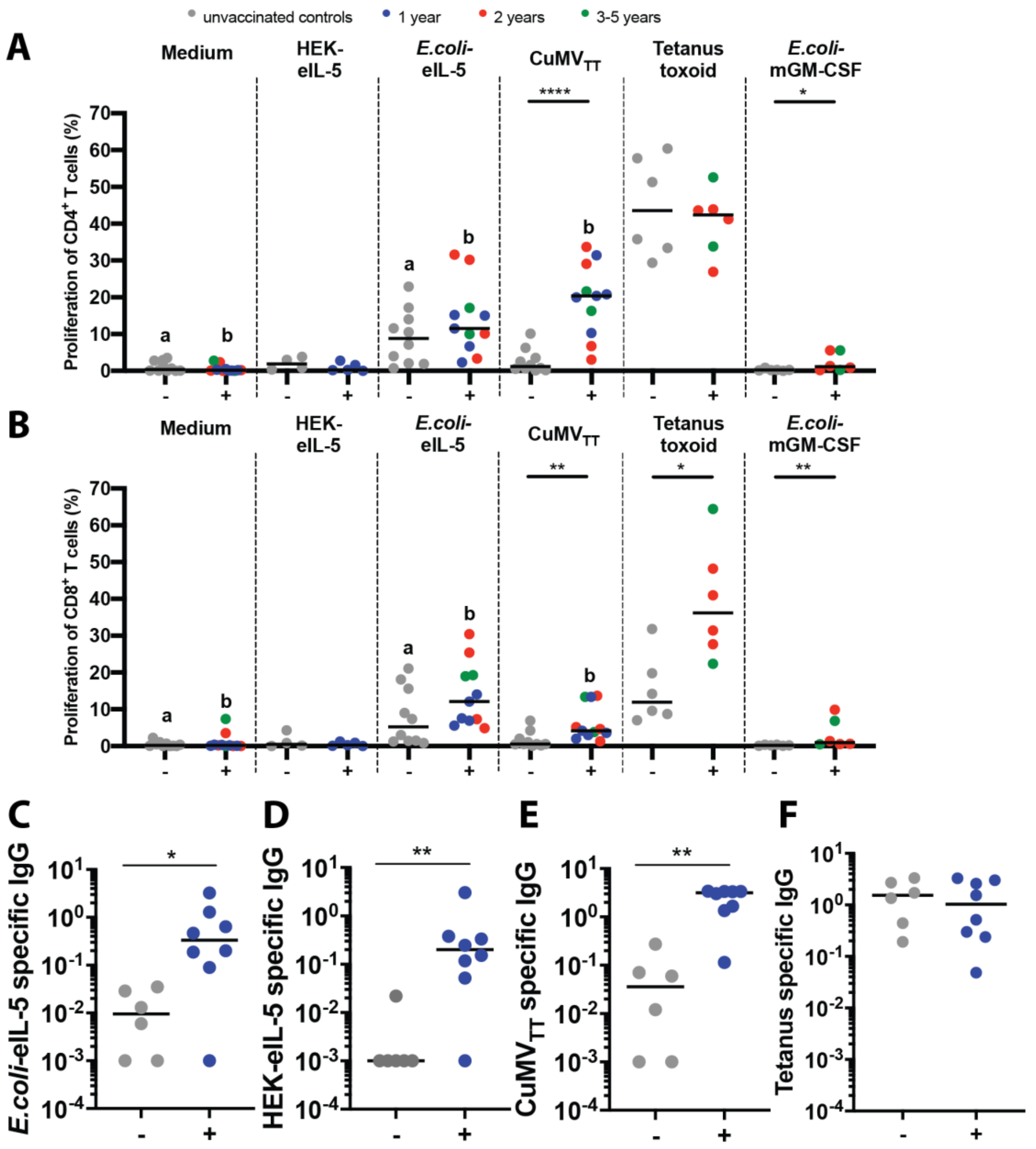

Figure 4. No auto-proliferative CD4⁺ and CD8⁺ T cells upon *in-vitro* re-stimulation of PBMCs, but specific IgGs upon resiquimod stimulated PBMCs. (A, B) Proliferation of CD4⁺ (A) and CD8⁺ (B) T cells upon *in-vitro* re-stimulation using medium control, HEK-eIL-5, *E.coli*-eIL-5, $CuMV_{TT}$, tetanus toxoid (TetaTox), or *E.coli*-mGM-CSF of PBMCs from unvaccinated (-) control horses (grey), first (blue), second (red), or third + (green) year vaccinated (+) horses. Values show individual horses and median. Differences between unvaccinated (-) controls and vaccinated (+) horses were analyzed with Mann-Whitney U test. Medium unstimulated cells were compared to stimulated cells by Wilcoxon matched-pairs rank with Bonferroni correction, significant differences between unstimulated and stimulated cells within groups indicated with a for controls and b for vaccinated horses. (C, D, E, F) Total IgG by resiquimod stimulated PBMCs from unvaccinated (-) and vaccinated (+) horses against *E.coli*-eIL-5 (C), HEK-eIL-5 (D), $CuMV_{TT}$ (E), tetanus toxoid (F) by ELISA. Values of individual horses with median on a log scale, statistical analysis by Mann-Whitney U test.

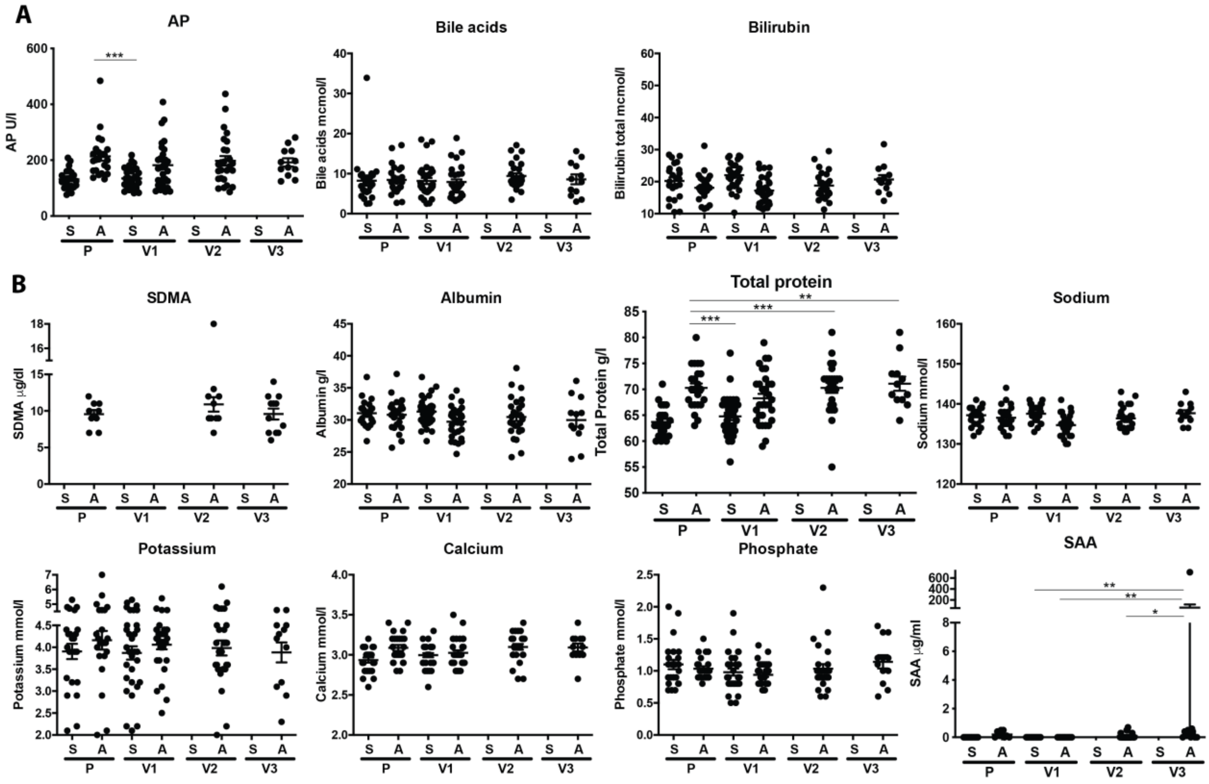

Figure 5. Additional liver and kidney parameters. Liver (**A**) and kidney (**B**) parameters were measured in the spring (S) and autumn (A) in the placebo year and 1st (V1), 2nd (V2) and 3rd (V3) treatment year. AP, alkaline phosphatase, SDMA, symmetric dimethylarginine, SAA, serum amyloid alpha. Values of individual horses with mean and SEM, statistical analysis by Kruskal-Wallis with Dunn's multiple comparison, statistical significance is only indicated when at least one value was outside of norm.

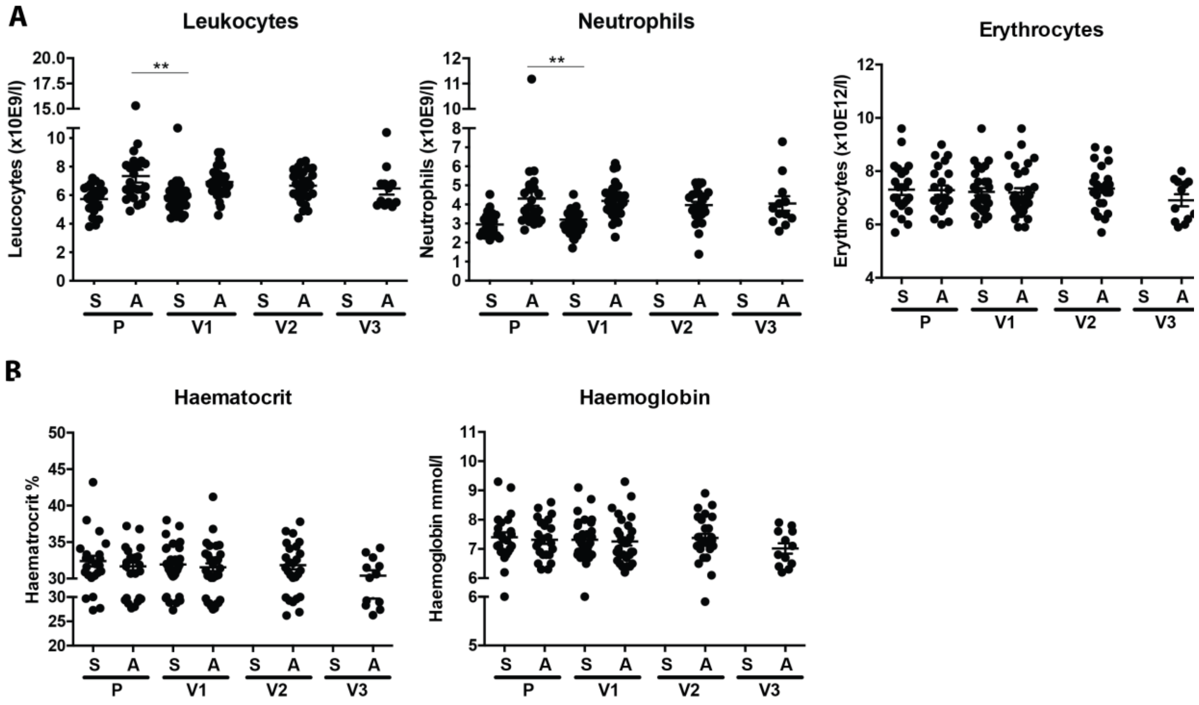

Figure 6. Blood differential count and hematology. Blood differential count (**A**) as well as analysis of hematocrit and hemoglobin concentration (**B**) was carried out in the spring (S) and autumn (A) in the placebo year and 1st (V1), 2nd (V2) and 3rd (V3) treatment year. Values of individual horses with mean and SEM, statistical analysis by Kruskal-Wallis with Dunn's multiple comparison, statistical significance is only indicated when at least one value was outside of norm.

© 2020 by the authors. Submitted for possible open access publication under the terms and conditions of the Creative Commons Attribution (CC BY) license (http://creativecommons.org/licenses/by/4.0/).
